# Correspondence: *SEMA4A* variation and risk of colorectal cancer

**DOI:** 10.1038/ncomms10611

**Published:** 2016-03-10

**Authors:** Ben Kinnersley, Daniel Chubb, Sara E. Dobbins, Matthew Frampton, Stephan Buch, Maria N. Timofeeva, Sergi Castellví-Bel, Susan M. Farrington, Asta Forsti, Jochen Hampe, Kari Hemminki, Robert M. W. Hofstra, Emma Northwood, Claire Palles, Manuela Pinheiro, Clara Ruiz-Ponte, Clemens Schafmayer, Manuel R. Teixeira, Helga Westers, Tom van Wezel, D. Timothy Bishop, Ian Tomlinson, Malcolm G. Dunlop, Richard S. Houlston

**Affiliations:** 1 Division of Genetics and Epidemiology, The Institute of Cancer Research, Surrey, Sutton SM2 5NG, UK; 2 Department of Medicine I, University Hospital Dresden, Dresden 23538, Germany; 3 Colon Cancer Genetics Group, Institute of Genetics and Molecular Medicine, University of Edinburgh and Medical Research Council (MRC) Human Genetics Unit, Edinburgh EH4 2XU, UK; 4 Department of Gastroenterology, Hospital Clínic, Centro de Investigación Biomédica en Red de Enfermedades Hepáticas y Digestivas, Institut d'Investigacions Biomèdiques August Pi i Sunyer, University of Barcelona, Catalonia, Barcelona 8036, Spain; 5 German Cancer Research Center, Heidelberg 69120, Germany; 6 Department of Internal Medicine I, Hospital Schleswig-Holstein, Kiel 24105, Germany; 7 Department of Clinical Genetics, Erasmus Medical Center, Rotterdam 3000 CA, The Netherlands; 8 University of Gronigen, University Medical Centre Gronigen, Department of Genetics, Gronigen 9700 RB, The Netherlands; 9 Section of Epidemiology and Biostatistics, Leeds Institute of Cancer and Pathology, University of Leeds, Leeds LS9 7TF, UK; 10 Molecular and Population Genetics Laboratory, Wellcome Trust Centre for Human Genetics, University of Oxford, Oxford OX3 7BN, UK; 11 Department of Genetics, Portugese Oncology Institute, Porto 4200-072, Portugal; 12 Galician Public Foundation of Genomic Medicine, Centro de Investigación Biomédica en Red de Enfermedades Rares, Genomics Medicine Group, Hospital Clínico, Santiago de Compostela, University of Santiago de Compostela, Galicia 15782, Spain; 13 Department of General and Thoracic Surgery, University Hospital Schleswig-Holstein, Kiel 24105, Germany; 14 Leiden Department of Pathology, Leiden University Medical Center, Leiden, 2333 ZA, The Netherlands

Sill and co-workers^1^ report that germline variation in semaphorin 4A (*SEMA4A*) influences colorectal cancer (CRC) risk. This stems from identifying the *SEMA4A* p.Val78Met variant in one kindred with familial colorectal cancer type X (FCCTX) and subsequently p.Gly484Ala (c.1451G>C, rs148744804) and p.Ser326Phe (c.977C>T) mutations along with the single nucleotide polymorphism (SNP) p.Pro682Ser (c.2044C>T, rs76381440) among an additional 53 FCCTX cases. In comparing the frequency of rs76381440 genotype in 47 FCCTX cases and 1,138 controls, c.2044T carrier status was reported to be associated with 6.79-fold increased CRC risk^1^.

Here, we report a well-powered study that casts doubt on *SEMA4A* as a CRC predisposition gene. This has important implications for clinical genetics because inappropriate screening or intervention might be recommended to carriers.

First, we studied the contribution of the recurrent variants, rs148744804 and rs76381440, to CRC analysing 6,856 CRC cases and 10,090 controls from six European populations as previously described^2^,^3^. These comprised (1) 3,666 English case patients (*n*=250 from the CORGI study; *n*=957 from the QUASAR study; *n*=1,168 from NSCCG; *n*=1,291 from a Leeds case–control series) and 6,140 control patients (*n*=5,694 from the 1958 Birth Cohort; *n*=446 from the Leeds series); (2) 2,052 Scottish CRC cases and 2,004 Scottish controls (*n*=1,452 from the 1935 and 1928 Lothian birth cohorts; *n*=552 from generation Scotland); (3) 276 Spanish cases and 284 controls; (4) 800 Dutch samples (*n*=337 Leiden cases and *n*=337 controls; *n*=74 Groningen cases and *n*=52 controls); (5) 199 Portuguese cases and 186 controls and (6) 1,339 German samples (*n*=77 Heidelberg cases and *n*=88 controls; *n*=175 Kiel cases and *n*=999 controls). Collectively these samples provide >99% power (*α*=0.05) to detect the lower limit of the point estimate reported by Sill and co-workers for the association between p.Pro682Ser and CRC^1^) (odds ratio [OR]=2.6).

We used Infinium HumanExome BeadChips (Illumina San Diego, CA) to genotype our samples as previously described^2^,^3^ and extracted the genotypes for rs148744804 and rs76381440. We validated genotyping by sequencing 541 random samples, providing very strong concordance (*r*^2^=1.0 and 0.99 for rs148744804 and rs76381440, respectively; Supplementary Table 1). We used principal component analysis to confirm ancestral comparability of cases and controls (Supplementary Figure 1).

None of the six series showed a statistically significant difference in frequency of rs148744804 or rs76381440 genotype between cases and controls (Table 1). In a meta-analysis of data from all studies, we found no association between c.1451C or c.2044T carrier status and CRC (OR=1.14, 95% confidence interval [CI]: 0.58–2.24, *P*_heterogenity_=0.87, *I*^2^=0% and OR=1.04, 95% CI: 0.89–1.22, *P*_heterogenity_=0.36, *I*^2^=9%, respectively; Fig. 1). Principal component analysis adjustment had no impact on findings.

Following on from these analyses we examined the mutational spectra of *SEMA4A* in 1,006 familial early-onset CRC cases (≥1 first-degree relative with CRC, <56yrs; 158 with FCCTX) from the National Study of Colorectal Cancer Genetics (NSCCG) and 1,609 1958 BC controls sequenced using Illumina Truseq exome capture in conjunction with HiSeq2000 technology (Supplementary Figure 2, Supplementary Table 2). Over 99% of the *SEMA4A* transcript was covered at a depth greater than 10 reads (average coverage 38 ×, Supplementary Figure 2). We identified 28 protein changing variants in 354 samples. Of these variants, there were three unique frameshifts present in two controls and one case. Overall, 13% of CRC cases (16% FCCTX) had a protein changing variant in *SEMA4A* in comparison with 14% of controls.

There are a number of possible explanations for the disparity between our findings and those reported by Sill and co-workers^1^. Population stratification can lead to spurious associations, and this is especially important with rare variants. The study by Sill and co-workers did not account for this and indeed in comparing the frequency of rs14874408 included cases from both the US and Germany, whereas we ensured ancestral comparability of case patients and control subjects from single nucleotide polymorphism genotypes, thereby excluding this as a source of bias. Generalisability is central to establishing a mutation–phenotype relationship. The evidence for p.Val78Met being causative in FCCTX is based on incomplete segregation in the family reported by Schulz *et al*. Hence there is the issue of type 1 error.

Much of the missing heritability of CRC is likely to be a result of high/moderate penetrance mutations and rare variants. As illustrated by the recent identification of *POLE* and *POLD1* as a cause of familial CRC^4^, this class of susceptibility is especially important in understanding cancer biology and for clinical practice. Hence there is a strong rationale for seeking to identify additional such genes. Given the high frequency of deleterious mutations carried by the healthy population, it is becoming increasingly clear that robust and well-powered studies are required to prevent erroneous findings from exome-sequencing projects being asserted to be causal of disease.

In conclusion, in this well-powered study, we find no evidence to support variation in *SEMA4A* as a determinant of CRC risk. Given that *a priori SEMA4A* is not a strong candidate CRC predisposition gene, having previously been shown to cause eye disease, we feel that caution should be exercised before *SEMA4A* is considered as a cause of CRC.

## Additional information

**How to cite this article**: Kinnersley, B. *et al*. Correspondence: *SEMA4A* variation and risk of colorectal cancer. *Nat. Commun*. 7:10611 doi: 10.1038/ncomms10611 (2016).

## Supplementary Material

Supplementary Information Supplementary Figures 1-2, Supplementary Tables 1-2 and Supplementary Methods.

## Figures and Tables

**Figure 1:**
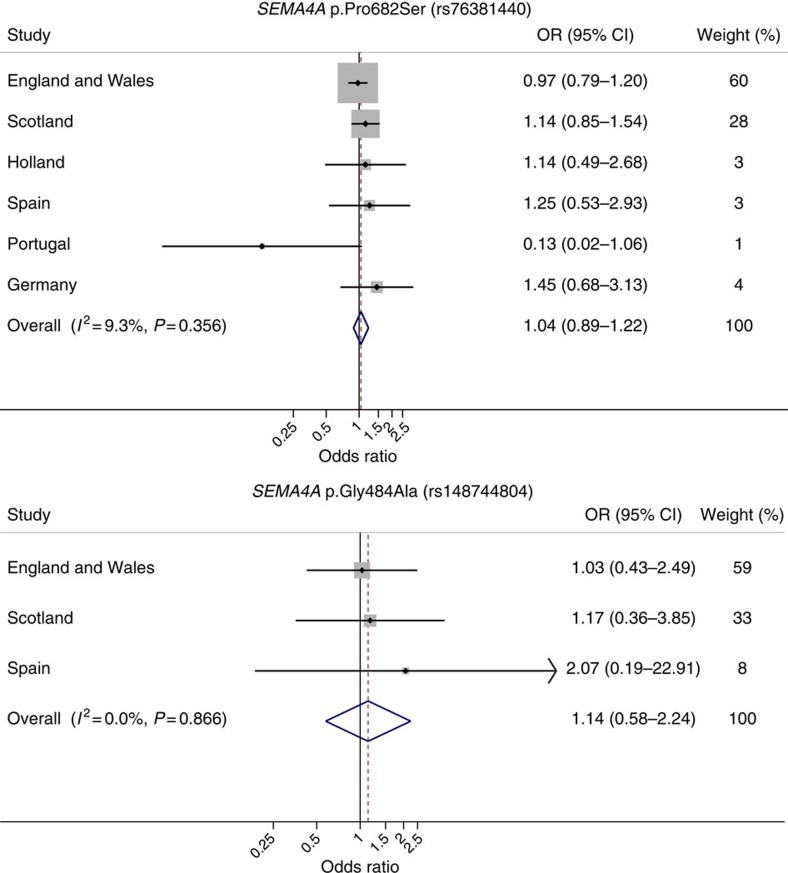
Forest plot of association between rs148744804 and rs76381440 *SEMA4A* genotypes and colorectal cancer risk. Studies were weighted according to the inverse of the variance of the log of the OR calculated by unconditional logistic regression. Meta-analysis under a fixed-effects model was conducted using standard methods. Cochran's *Q* statistic to test for heterogeneity and the *I*^*2*^ statistic to quantify the proportion of the total variation due to heterogeneity were calculated. Horizontal lines indicate 95% confidence intervals (CIs). Boxes indicate odds ratio (OR) point estimate; its area is proportional to the weight of the study. Diamond (and broken line) indicates overall summary estimate, with CI given by its width. Unbroken vertical line indicates null value (OR=1.0).

**Table 1:** *SEMA4A* rs76381440 (p.Pro682Ser, c.2044C>T) and rs148744804 (p.Gly484Ala, c.1451G>C) genotype counts and association statistics for the six colorectal cancer case–control studies.

Study	Case					Control					OR (95% CI)	*P*
	AA	AB	BB	Total	MAF	AA	AB	BB	Total	MAF		
*(A) rs76381440*												
England and Wales	0	152	3,514	3,666	0.021	2	259	5,879	6,140	0.021	0.97 (0.79–1.20)	0.80
Scotland	0	99	1,953	2,052	0.024	1	84	1,919	2,004	0.021	1.14 (0.85–1.54)	0.37
Holland	0	12	399	411	0.015	0	10	380	390	0.013	1.14 (0.49–2.68)	0.76
Spain	0	12	264	276	0.022	0	10	274	284	0.018	1.25 (0.53–2.93)	0.62
Portugal	0	1	198	199	0.003	0	7	179	186	0.019	0.13 (0.02–1.06)	0.06
Germany	0	9	243	252	0.018	0	27	1,060	1,087	0.012	1.44 (0.68–3.13)	0.34
Combined	0	285	6,571	6,856	0.021	3	397	9,691	10,091	0.020	1.04 (0.89–1.22)	0.63
												
*(B) rs148744804*												
England/Wales	0	8	3,658	3,666	0.0011	0	13	6,127	6,140	0.0011	1.03 (0.43–2.49)	0.95
Scotland	0	6	2,046	2,052	0.0015	0	5	1,999	2,004	0.0012	1.17 (0.36–3.85)	0.79
Holland	0	0	411	411	0.0000	0	1	389	390	0.0013	NA	NA
Spain	0	2	274	276	0.0036	0	1	283	284	0.0018	2.07 (0.19–22.9)	0.56
Portugal	0	1	198	199	0.0025	0	0	186	186	0.0000	NA	NA
Germany	0	0	252	252	0.0000	0	0	1,087	1,087	0.0000	NA	NA
Combined	0	17	6,839	6,856	0.0012	0	20	10,071	10,091	0.0010	1.14 (0.58–2.24)	0.71

NA=not applicable; MAF=minor allele frequency; OR=odds ratio; CI=confidence interval. AA, AB and BB denote minor homozygote, heterozygote and major homozygote genotypes respectively.

Meta-analysis: *P*_het_=0.36, *I*^2^=9.3%. Meta-analysis: *P*_het_=0.87, *I*^2^=0%.
