# Supplementary material for: Correspondence: SEMA4A variation and risk of colorectal cancer
Source: Nat Commun. 2016 Mar 10;7:10611. doi: 10.1038/ncomms10611 (PMC4792923; doi:10.1038/ncomms10611)
Supplement: Supplementary Information — Supplementary Figures 1-2, Supplementary Tables 1-2 and Supplementary Methods. [file ncomms10611-s1.pdf]

Supplementary Figure 1: Principal component analysis of cases and controls in the different cohorts genotyped on the exome array. The first two principal components of the analysis were plotted. Case-control cohorts plotted with HapMap populations (CEU; Utah residents with Northern and Western European ancestry, CHB; Han Chinese in Bejing, China, JPT; Japanese in Tokyo, Japan, YRI; Yoruba in Ibadan, Nigeria).

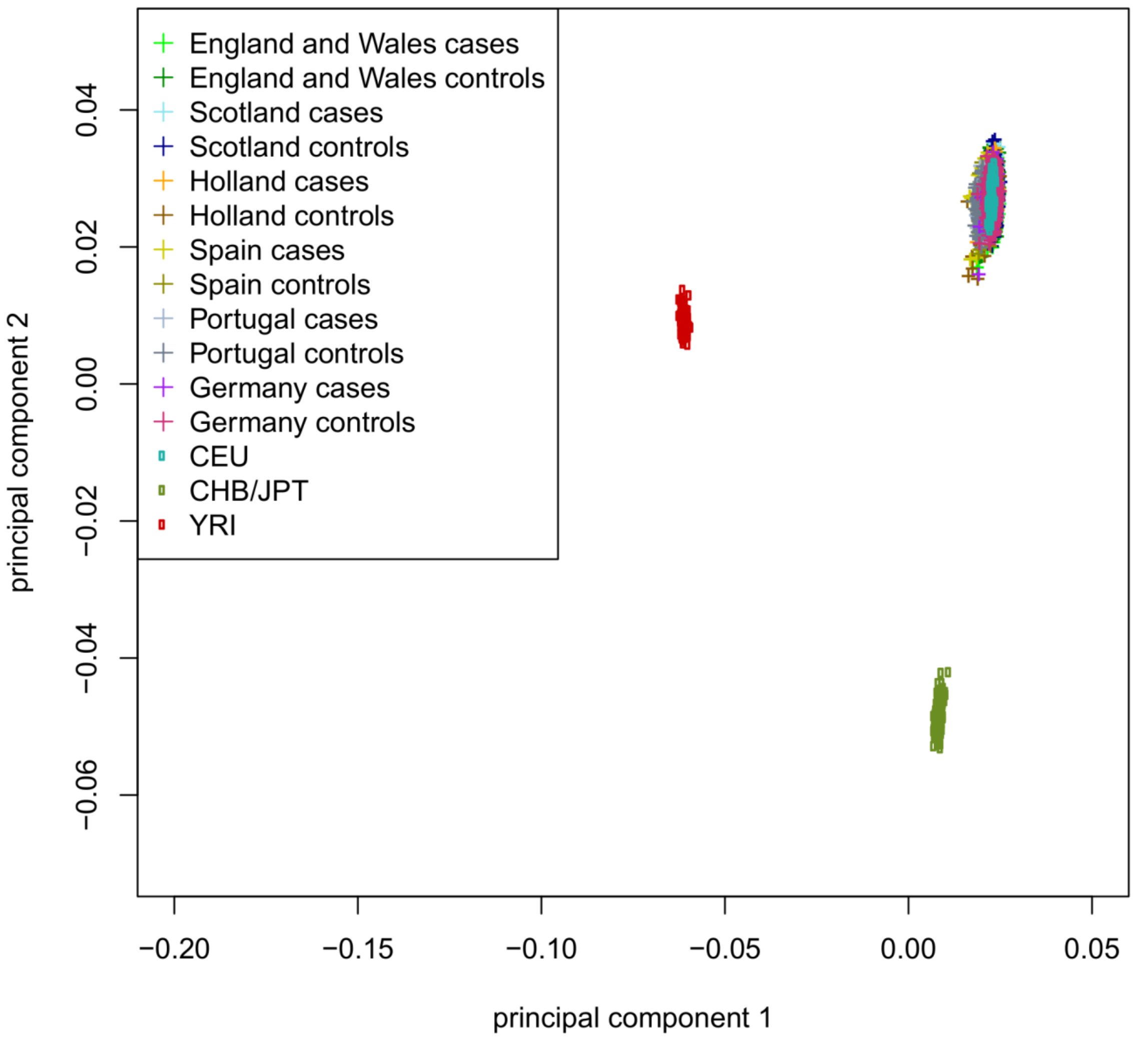

Supplementary Figure 2: Protein changing variants present in SEMA4A alongside the average coverage at each position. Exons are highlighted in alternating red and grey and UTRs are white.

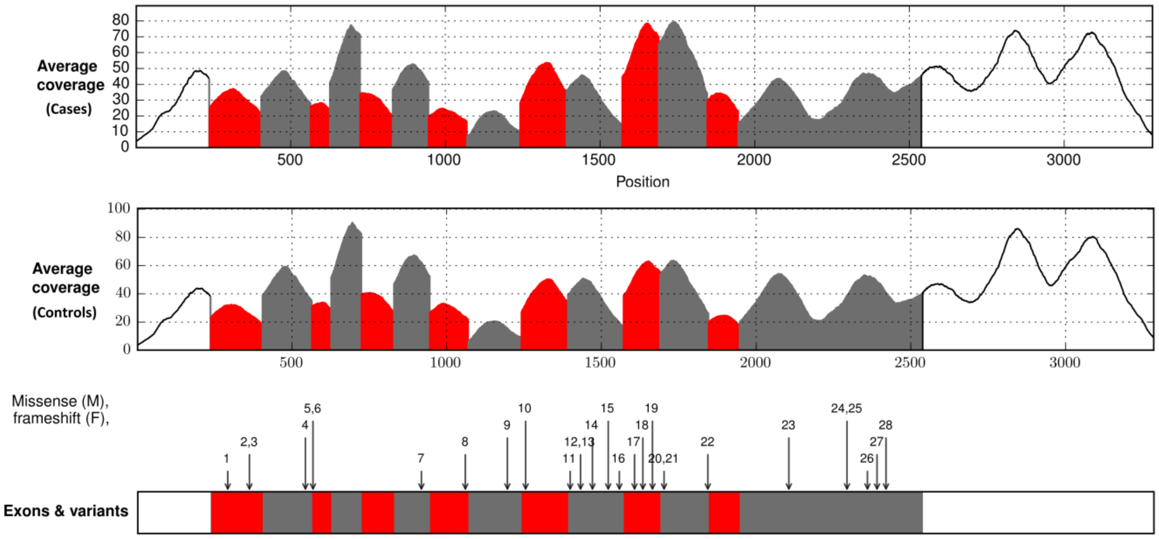

1: c.27delN, p.Trp11GlyfsTer59, (F)
2: c.95N>T, p.Gly32Val, (M)
3: c.97N>A, p.Gly33Arg, (M)
4: c.278N>C, p.Gly93Ala, (M)
5: c.302N>C, p.Ile101Thr, (M)
6: c.305N>T, p.Pro102Leu, (M)
7: c.657N>C, p.Lys219Asn, (M)
8: c.800N>A, p.Arg267Lys, (M)
9: c.937N>T, p.Asp313Tyr, (M)
10: c.997N>G, p.Thr333Ala, (M)
11: c.1143delN, p.Gly382AlafsTer8, (F)
12: c.1175N>C, p.Met392Thr, (M)
13: c.1176N>A, p.Met392Ile, (M)
14: c.1214N>T, p.Thr405Met, (M)
15: c.1265N>T, p.Thr422Ile, (M)
16: c.1301N>C, p.Met434Thr, (M)
17: c.1351N>A, p.Asp451Asn, (M)
18: c.1378_1379insT, p.Gln461SerfsTer5, (F)
19: c.1409N>A, p.Arg470His, (M)
20: c.1444N>A, p.Phe482Ile, (M)
21: c.1451N>C, p.Gly484Ala, (M)
22: c.1589N>G, p.Asn530Ser, (M)
23: c.1853N>G, p.Val618Gly, (M)
24: c.2038N>C, p.Tyr680His, (M)
25: c.2044N>T, p.Pro682Ser, (M)
26: c.2107N>A, p.Val703Met, (M)
27: c.2138N>A, p.Arg713Gln, (M)
28: c.2167N>T, p.Arg723Cys, (M)

Supplementary Table 1: Validation of rs76381440 and rs148744804 genotypes in 541 random samples by sequencing. (a) rs76381440; (b) rs148744804. Concordant and discordant calls are coloured green and yellow respectively. NC, no-call.

A) rs76381440

		Exome sequence genotype calls			
		TT	TC	CC	NC
Exome array	TT	1	0	0	0
Genotype	TC	1	32	0	0
Calls	CC	0	0	503	5
	NC	0	0	0	0

B) rs148744804

		Exome sequence genotype calls			
		CC	CG	GG	NC
Exome array	CC	0	0	0	0
Genotype	CG	0	0	0	0
Calls	GG	0	0	541	1
	NC	0	0	0	0

Supplementary Table 2: The number of NSCCG CRC cases and 1958BC controls containing protein changing variants in *SEMA4A*. Frameshift variants are highlighted in red. Variant number relates to number in Supplementary Figure 2.

Variant number	Protein Change	Familial CRC Cases			Controls
		Non-FCCTX	FCCTX	All cases	
1	p.Trp11GlyfsTer59	0	0	0	1
2	p.Gly32Val	0	0	0	1
3	p.Gly33Arg	1	0	1	0
4	p.Gly93Ala	1	0	1	0
5	p.Ile101Thr	0	0	0	1
6	p.Pro102Leu	0	0	0	1
7	p.Lys219Asn	1	0	1	0
8	p.Arg267Lys	1	0	1	0
9	p.Asp313Tyr	0	1	1	0
10	p.Thr333Ala	0	0	0	1
11	p.Gly382AlafsTer8	0	1	1	0
12	p.Met392Thr	0	0	0	1
13	p.Met392Ile	0	0	0	1
14	p.Thr405Met	0	0	0	1
15	p.Thr422Ile	2	0	2	1
16	p.Met434Thr	5	0	5	1
17	p.Asp451Asn	0	0	0	1
18	p.Gln461SerfsTer5	0	0	0	1
19	p.Arg470His	0	0	0	1
20	p.Phe482Ile	0	1	1	0
21	**p.Gly484Ala**	**1**	**1**	**2**	**0**
22	p.Asn530Ser	0	0	0	1
23	p.Val618Gly	0	0	0	1
24	p.Tyr680His	2	0	2	1
25	**p.Pro682Ser**	**31**	**9**	**40**	**70**
26	p.Val703Met	1	0	1	0
27	p.Arg713Gln	56	15	71	142
28	p.Arg723Cys	0	0	0	1

Supplementary Methods

Samples for exome sequencing

Cases: This comprised 1,006 unrelated patients with histologically-proven colorectal cancer (CRC; ICD9 codes 153 and 154) aged ≤55 years at diagnosis (mean age 48.7, standard deviation (SD)=6.0) which had at least one first-degree relative (FDR) with CRC, ascertained between 2003-2011 through the National Study of Colorectal Cancer Genetics (NSCCG)[1]. All the patients were UK residents and had self-reported European-Ancestry. Germline DNA was isolated from EDTA-venous bloods using standard methods and picogreen quantified. 153 out of 1,006 patients were defined as having familial colorectal cancer type X (FCCTX) due to meeting Amsterdam II criteria and not carrying a protein truncating variant or missense variant defined as pathogenic/likely pathogenic by InSight or

ClinVar[2] in the known CRC genes (*i.e. MLH1*, *MSH2*, *MSH6*, *PMS2*, *APC*, *MUTYH*, *SMAD4*, *BMPR1A*) as well as the exonuclease domains of *POLE* and *POLD1*. Written informed consent was obtained from all individuals with ethical review board approval (Multi-Research Ethics Committee 02/0/097) and the study was conducted in accordance with the declaration of Helsinki.

Controls: This comprised 1,609 healthy individuals from the UK 1958 Birth cohort (1958BC)[3] - 961 from the ICR1000 dataset (EGAD00001001021)[17] and an additional 648 1958BC individuals all sequenced at The Institute of Cancer Research as *per* the Discovery cases.

Exome sequencing

Sequencing protocol: 1ug of DNA from each individual was fragmented using a Covaris E Series instrument (Covaris Inc. Woburn, Massachusetts, USA). Indexed paired-end libraries were prepared using Illumina TruSeq 62Mb expanded exome enrichment kit, according to the manufacturer's instructions (Illumina, San Diego, CA, USA); 2x100bp sequencing was performed using Illumina HiSeq2000 technology.

Read mapping and variant analysis: Paired end fastq files were extracted using CASAVA software (v.1.8.1, Illumina) and aligned to build 37 (hg19) of the human reference genome using Stampy and BWA software[4]. Alignments were processed using the Genome Analysis Tool Kit (GATKv3) pipeline according to best practices[5,6]. Analysis was restricted to capture regions defined in the Truseq 62Mb bed file plus 100bp padding. The Variant Effect Predictor (VEP)[7] was used to provide annotations on the predicted impact of each variant together with functional classifications using CONDEL[8].

Exome array genotyping

Genotyping was performed using the Illumina HumanExome 12v1_A Beadchip array (Illumina, San Diego, CA, USA) in accordance with the manufacturer's recommendations as previously described[9,10]. Cluster boundaries were determined by calling study samples simultaneously.

SUPPLEMENTARY REFERENCES

1. Penegar, S. *et al.* National study of colorectal cancer genetics. *Br J Cancer* **97**, 1305-9 (2007).
2. Landrum, M.J. *et al.* ClinVar: public archive of relationships among sequence variation and human phenotype. *Nucleic Acids Res* **42**, D980-5 (2014).
3. Power, C. & Elliott, J. Cohort profile: 1958 British birth cohort (National Child Development Study). *Int J Epidemiol* **35**, 34-41 (2006).
4. Lunter, G. & Goodson, M. Stampy: a statistical algorithm for sensitive and fast mapping of Illumina sequence reads. *Genome Res* **21**, 936-9 (2011).

5. McKenna, A. *et al.* The Genome Analysis Toolkit: a MapReduce framework for analyzing next-generation DNA sequencing data. *Genome Res* **20**, 1297-303 (2010).
6. DePristo, M.A. *et al.* A framework for variation discovery and genotyping using next-generation DNA sequencing data. *Nat Genet* **43**, 491-+ (2011).
7. McLaren, W. *et al.* Deriving the consequences of genomic variants with the Ensembl API and SNP Effect Predictor. *Bioinformatics* **26**, 2069-70 (2010).
8. Gonzalez-Perez, A. & Lopez-Bigas, N. Improving the assessment of the outcome of nonsynonymous SNVs with a consensus deleteriousness score, Condel. *Am J Hum Genet* **88**, 440-9 (2011).
9. Kinnersley, B. *et al.* Re: Role of the Oxidative DNA Damage Repair Gene OGG1 in Colorectal Tumorigenesis. *Journal of the National Cancer Institute* (2014).
10. Timofeeva, M.N. *et al.* Recurrent Coding Sequence Variation Explains Only A Small Fraction of the Genetic Architecture of Colorectal Cancer. *Sci Rep* **5**, 16286 (2015).
